# The *Mtb*-HIV syndemic interaction: why treating *M. tuberculosis* infection may be crucial for HIV-1 eradication

**DOI:** 10.2217/fvl-2019-0069

**Published:** 2020-03-24

**Authors:** Robyn Waters, Mthawelanga Ndengane, Melissa-Rose Abrahams, Collin R Diedrich, Robert J Wilkinson, Anna K Coussens

**Affiliations:** 1 Wellcome Centre for Infectious Diseases Research in Africa, Institute of Infectious Disease & Molecular Medicine, University of Cape Town, Observatory 7925, WC, South Africa; 2 Department of Medicine, University of Cape Town, Observatory 7925, WC, South Africa; 3 Department of Pathology, University of Cape Town, Observatory 7925, WC, South Africa; 4 Department of Pediatrics, Children’s Hospital of Pittsburgh of the University of Pittsburgh Medical Center, Pittsburgh, PA, USA; 5 Department of Infectious Diseases, Imperial College London, London W2 1PG, United Kingdom; 6 The Francis Crick Institute, London NW1 1AT, United Kingdom; 7 Infectious Diseases and Immune Defence Division, The Walter & Eliza Hall Institute of Medical Research, Parkville 3279, VIC, Australia; 8 Division of Medical Biology, Faculty of Medicine, Dentistry & Health Sciences, University of Melbourne, Parkville 3279, VIC, Australia

**Keywords:** AIDS, granuloma, HIV-1 cure, immune activation, latency, transmission, tuberculosis, viral expansion, viral reservoir

## Abstract

Accelerated tuberculosis and AIDS progression seen in HIV-1 and *Mycobacterium tuberculosis* (*Mtb*)-coinfected individuals indicates the important interaction between these syndemic pathogens. The immunological interaction between HIV-1 and *Mtb* has been largely defined by how the virus exacerbates tuberculosis disease pathogenesis. Understanding of the mechanisms by which pre-existing or subsequent *Mtb* infection may favor the replication, persistence and progression of HIV, is less characterized. We present a rationale for the critical consideration of ‘latent’ *Mtb* infection in HIV-1 prevention and cure strategies. In support of this position, we review evidence of the effect of *Mtb* infection on HIV-1 acquisition, replication and persistence. We propose that ‘latent’ *Mtb* infection may have considerable impact on HIV-1 pathogenesis and the continuing HIV-1 epidemic in sub-Saharan Africa.

## Graphical abstract

The proposed impact of *Mycobacterium tuberculosis* (*Mtb*) coinfection (solid lines) on the life-course progression of HIV-1 infection (dotted lines). In addition to enhancing the speed of AIDS progression and tuberculosis development in those HIV-1 infected, *Mtb* infection increases myeloid and T-cell immune activation and the susceptibility of cells to HIV-1 infection and HIV-1 replication in infected and bystander cells. Following antiretroviral therapy initiation, a larger pool of reservoir cells harboring HIV-1 and higher immune activation status has the potential to result in more frequent blips in viremia above the level of clinical detection (thin line).

By the end of 2018, an estimated 37.9 million people worldwide were living with HIV, around 95% infected with HIV-1 and about 13 million HIV-infected persons are estimated to be coinfected with *Mycobacterium tuberculosis* (*Mtb*); although 49% are unaware of their coinfection status and thus are not receiving appropriate care [1,2]. The introduction of antiretroviral therapy (ART) has decreased HIV-1 mortality, transmission and the progression to AIDS, and progressively decreased rates of new HIV-1 cases annually. However, the incidence of new infections is rising in almost 50 countries [3] and there were still 1.7 million new infections in 2018 [2]. Despite the extension of life and reduced morbidity that ART provides to those living with HIV-1, ART remains a life-long necessity. Better ways to prevent HIV-1 transmission, limit HIV-1 progression and cure infection are urgently needed.

Active tuberculosis (TB) is the greatest cause of mortality in HIV-1 infected individuals, causing approximately a third of HIV-1 associated deaths and is a leading cause of HIV-1 progression to AIDS [4]. HIV-infected patients can have a 5–10% annual risk of developing TB, compared with a 5–15% life-time risk for HIV-1 uninfected persons [5]. In 2018, of 10 million cases of TB, HIV-coinfected persons represented 8.6% of cases and 17% of TB-associated deaths. Africa accounts for 71% of all HIV-TB cases and 84% of coinfection deaths [4]. Of the reported HIV-TB cases, despite 86% of cases reported to be on ART, death from TB remains more common compared with those HIV-uninfected [4]. Increased risk of mortality is associated with advanced immunodeficiency, chronic immune activation, increased disease dissemination and severity and co-morbid opportunistic infections. This accelerated TB disease progression and increased risk of mortality indicates a critical interaction between these two syndemic pathogens [5,6].

TB incidence rates are falling in people living with HIV, due to earlier ART initiation [7], but there remains an enormous burden of untreated latent *Mtb* in the community [8]. Recent advances in our understanding of how both active and latent *Mtb* infection can contribute to HIV-1 viral expansion have encouraged new interest in the contribution of *Mtb* infection to HIV-1 progression. In this review, we build an evidence-based argument surrounding the epidemiological, cellular and molecular basis as to how latent *Mtb* infection (LTBI) may contribute to HIV-1 disease progression. We investigate each step in the HIV-1 life cycle and present evidence to support a role of *Mtb* in enhancing or blocking each step (Table 1). We conclude with a discussion on the important factors, which may impact HIV-1 prevention and cure strategies.

**Table 1.** Potential cellular mechanisms which increase HIV-1 infection, replication and reservoir site expansion, modified by *Mycobacterium tuberculosis* infection and the consequences on HIV-1 infection course.

*Mtb*-induced effects on	HIV-1 infection	HIV-1 replication	Cellular transmigration	Immune evasion
Cellular responses	Increased CCR5 and CXCR4 surface presentation on *Mtb*-antigen-specific CD4^+^ T-cells and increased CD38^+^/HLA-DR^+^ T-cells	Increased proinflammatory cytokine environment (e.g., TNF, IL-1β, IL-6) induces HIV-1 LTR transcription	Increased CCL2 recruitment of HIV-infected CD16^+^ monocytes to peripheral sites of *Mtb* infection, transporting HIV-1 to *Mtb* microenvironment	Increased numbers of HIV-1-infected myeloid cells resistant to apoptosis
	Increased CCL3, CCL4, CCL5 secretion may block HIV-1 gp120 access to CCR5 inhibiting R5 infection	Increased secreted CCL5 enhances X4 HIV-1 replication	Increased CXCL10 recruitment of HIV-1-infected T-cells to *Mtb* microenvironment	Impaired NK cell IFN-γ production and reduced ADCC (not confirmed in context of coinfection)
	Increased CCR5 and CXCR4 on mononuclear cells, increased CXCR4 on alveolar macrophages and increased CD16^+^CD4^+^ monocytes	Coinfected myeloid cells increase HIV-1 replication in autocrine manner	*Mtb*-infected APC migration to HIV-1-infected LN, creating coinfection microenvironment	Impaired APC cross-presentation to CD8^+^ T-cells reducing CTL killing (not confirmed in context of coinfection)
	Increased cell-to-cell spread via APC and *Mtb*-induced macrophage nanotubes	Cytokine production by *Mtb*-infected cell can induce replication in bystander HIV-1-infected APC and T-cells, in paracrine manner	TB granuloma creates ideal cellular architecture for dynamic cellular migration and cell–cell interactions between LN, blood and tissue	Increased complement and FcγR expression by circulating monocytes and neutrophils may modify the diversity of HIV-1 antibody responses (yet to be confirmed)
Acute HIV-1 infection	Increased pool of susceptible cells increasing the likelihood of establishing HIV-1 infection	Increased replication and higher peak viral titre, increasing risk of transmission and faster spread of infection	Larger migration of infected cells seeding more lymphoid tissues increasing the viral set point	Impact on antigen cross-presentation and early CD8^+^ CTL killing remains to be determined
Chronic HIV-1 infection	Faster depletion of CD4^+^ T-cells and faster AIDS progression	Higher VL, enhanced replication of emergent X4-tropic virions and increased risk of transmission	Larger number of HIV-1 reservoirs seeded in different tissue resident myeloid cells	Larger number of myeloid cells, which can become HIV-1 reservoirs
HIV-1 during long-term ART	Cell-to-cell spread via HIV-1-infected myeloid cells will continue to expand infection despite plasma viral suppression	Continued viral replication in myeloid reservoir cells, contributing to chronic innate cell activation	Enhanced reconstitution of CD27^+^CD4^+^ central memory T-cells recruited to sites of *Mtb* infection	Larger pool and diversity of reservoir cells requiring different targeted strategies for HIV-1 elimination

ADCC: Antibody-dependent cellular cytotoxicity; APC: Antigen-presenting cell; ART: Antiretroviral therapy; CTL: Cytolytic T lymphocyte; FcγR: Fc gamma receptor; LN: Lymph node; LTR: Long terminal repeat; *Mtb*: *Mycobacterium tuberculosis*; NK: Natural killer; TB: Tuberculosis; VL: Viral load; X4, CXCR4.

## Evolution of the world’s deadliest syndemic infection

**Figure 1.**
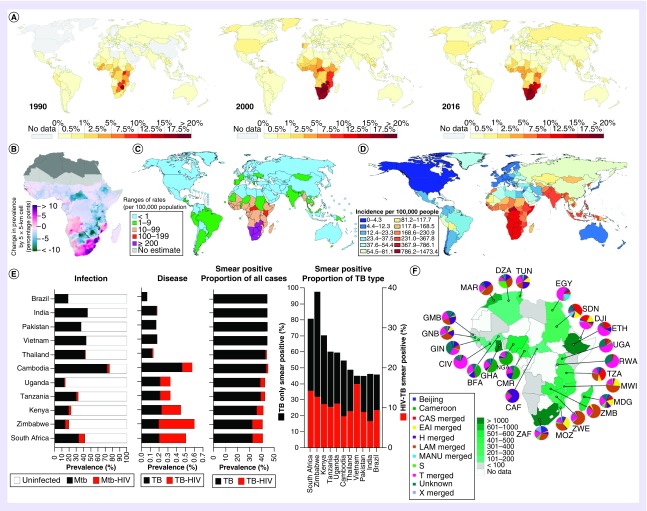
Epidemiological relationship between HIV-1 prevalence and tuberculosis incidence and infection from 1990 to 2017. **(A)** Prevalence of HIV-1 in adults aged 15–49, from 1990 to 2016. **(B)** Change in HIV-1 prevalence in adults aged 15–49 from 2000 to 2017 (countries in dark gray were not included in the analysis, grid cells with fewer than ten people per 1 × 1 km and classified as barren or sparsely vegetated, are colored light gray). **(C)** Estimated numbers of HIV-TB cases per 100,000 population (all ages) in 2000. **(D)** Age-standardized TB cases (excluding HIV) per 100,000 population (all ages) in 2016. **(E)** Prevalence of latent *Mtb* and *Mtb*-HIV-1 infection, TB and TB-HIV-1 disease, proportion of smear positivity attributed to TB or TB-HIV-1 and the proportion of TB (left Y-axis) and TB-HIV-1 (right Y-axis) cases smear positive in 2000, prior to HIV-1 expansion. **(F)** Proportion of *Mtb* lineages represented across African countries in pie charts. Euro-American Lineage 4 LAM strain (brown) is found most commonly in southern African countries, including those with the greatest increase in HIV-1 prevalence between 2000–2017 **(B):** MOZ and ZAF country codes (www.worldatlas.com/aatlas/ctycodes.htm). **(A)** Source: UNAIDS World Bank, OurWorldInData.org/hiv-aids/ [15,16]. **(B)** Reproduced with permission from [9]. **(C)** Reproduced with permission from [17] © American Medical Association (2003). All rights reserved. **(D)** Reproduced with permission from [10]. **(E)** Tabulated data extracted from [17] are replotted. Reproduced with permission from [17] © American Medical Association (2003). All rights reserved. **(F)** Reproduced with permission from [18]. LAM: Latin American Mediterranean; MOZ: Mozambique; *Mtb: Mycobacterium tuberculosis*; TB: Tuberculosis; ZAF: South Africa.

HIV-1 was the primary cause of the increase of the TB epidemic in sub-Saharan Africa, where the global burden of HIV-1 is concentrated (Figure 1) [5,9]. Although there was a significantly high incidence of TB in sub-Saharan Africa, the HIV-1 epidemic caused up to a tenfold increase in TB incidence [9]. A comparison between 2016 HIV-1 prevalence across Africa and non-HIV-1 TB incidence indicates that increasing prevalence of HIV-1 infection is the greatest in countries with the highest non-HIV-1-associated TB incidence (Figure 1B–D) [9,10]. HIV-TB is predicted to transmit less than non-HIV-1 TB [11], due to the lower proportion of smear positive TB cases (Figure 1F) and their faster time to disease progression, reducing the infectious period of HIV-TB patients. During the early expansion of the HIV-1 epidemic, there was no consistent and substantial increase in TB transmission to HIV-uninfected persons, supporting the limited contribution of increasing HIV-associated TB to the annual rate of *Mtb* infection [12–14].

Countries in Africa with the highest HIV-1 burden have a higher proportion of non-HIV-1-associated TB cases that are smear positive and to a lesser extent this extends to those HIV-1-coinfected (Figure 1E) [17]. This indicates a higher risk of *Mtb* transmission in the absence of HIV-1 and a high incidence of LTBI. Moreover, in TB high-burden settings, up to 50% of HIV-uninfected youth have LTBI by 15–17 years [19], suggesting, excluding mother to child transmission, *Mtb* infection is more likely to occur prior to HIV-1 acquisition.

A further consideration to the contribution of LTBI to HIV-1 progression is the geographical distribution of *Mtb* strains across Africa, with strains of differing lineages varying in the inflammatory phenotype they induce in infected phagocytes [20]. Southern Africa countries with the highest HIV-1 prevalence show the greatest proportion of *Mtb* arising from the Euro-American Lineage 4 LAM clade (Figure 1F) [18]. Given the inflammatory phenotype of *Mtb* strains have been associated with differing capacity to induce HIV-1 replication in peripheral blood cells, *in vitro* [21,22], the prevalence of varying strain types within a population may further exacerbate HIV-1 progression.

From the expansion of the syndemic during the 1990s, the rate of coinfection has continued to increase. Globally, in 2000, given the estimate of a third of the world with LTBI [23], an estimated 0.36% of the world’s population was *Mtb*-HIV-1 coinfected [17]. However, this rate varied significantly between regions, with HIV-1 high-burden countries in Africa noting coinfection rates of 1–8% (Figure 1E) [17]. A recent meta-analysis of global *Mtb* exposure assessed by interferon gamma release assays (IGRA) and tuberculin skin test (TST) reactivity rates, between 2005 and 2018, which supported a reduction in the global LTBI prevalence to a quarter of the world’s population, excluded an analysis of those HIV-coinfected [24]. Therefore, more recent global estimates of latent *Mtb*-HIV coinfection cannot be presented. However, this study demonstrated a strong correlation between TB case incidence and community level LTBI prevalence.

Together, these data indicate that the HIV-1 epidemic has continued to expand, despite ART rollout, in countries with the highest prevalence of non-HIV associated TB and that high rates of LTBI exist in countries with high TB case load. Given that HIV-1 coinfection is not associated with significantly increased TB transmission, we therefore forward a reverse causality hypothesis between *Mtb* and HIV-1 infection, whereby high rates of LTBI in a community contribute to exacerbating the HIV-1 epidemic.

Those that are HIV-1 infected are at increased risk of both reactivation of latent TB infection, during early HIV-1 and rapid progression following new *Mtb* infection, with greater risk of extrapulmonary TB as immune deficiency progresses [25,26]. ART decreases the incidence of TB among HIV-1 infected patients in countries with low or high TB incidence. A meta-analysis of nine observational cohort studies in which 37,879 patients were enrolled showed that ART reduced the incidence of TB by 67% [27], with an estimated prevention of 1.9 million HIV-TB cases by ART in 12 African countries, between 2003 and 2016 [7].

While there has been increased effort to control HIV-related TB in areas of high HIV-1 transmission, there has been less consideration regarding the impact of high community prevalence of LTBI on the HIV-1 epidemic in Africa, which has the potential to impact early HIV-1 progression following viral acquisition. Furthermore, continual community exposure to *Mtb* and recurrent infection could compound the increased risk of HIV-1 progression. If *Mtb* infection, therefore, has the potential to exacerbate HIV-1 acquisition and progression, latent *Mtb* elimination in HIV-1 high-burden settings may be an important factor to consider for HIV-1 prevention, treatment and cure strategies.

### Is Mtb or HIV-1 the greater syndemic driver?

**Figure 2.**
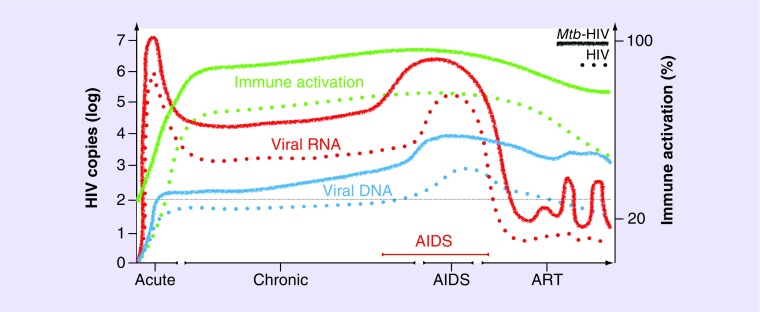
Representation of the proposed impact of *Mycobacterium tuberculosis* coinfection (solid lines) on the life-course progression of HIV-1 infection (dotted lines). A higher starting immune activation state during *Mtb* infection may lead to a higher peak in viral RNA (red) and infected cells with integrated viral DNA (blue) during the first few months of acute infection. As HIV-1 infection progresses over years, higher immune activation (green) persists and may result in a higher viral set point threshold and expansion of HIV-1 reservoirs cells containing HIV-1 DNA. With increased HIV-1 replication at tissue sites of *Mtb* infection and due to a higher proinflammatory environment and cellular activation, HIV-1 will progress faster into AIDS (red) and potential TB disease. TB will coincide with a secondary viremia peak and further expansion of infected cells with integrated DNA. Following ART initiation, a larger pool of reservoir cells harboring HIV-1 and higher immune activation status has the potential to result in more frequent blips in viremia above the level of clinical detection (thin line), following viral suppression and tissue-specific HIV-1 reservoirs persisting with cell-to-cell spread, rather than a slow reduction in cells harboring integrated viral DNA. ART: Antiretroviral therapy; *Mtb*: *Mycobacterium tuberculosis*; TB: Tuberculosis.

Active TB is a primary precipitate of AIDS progression [28]. Multiple prospective and retrospective studies demonstrate that peripheral HIV-1 RNA concentration (viral load [VL]) increases 1–2 log-fold in those HIV-1 infected at the time of TB diagnosis, and that HIV-1 VL decreases in some individuals, following successful TB treatment (Figure 2) [28–31]. This supports the posit that the inflammatory state during active TB enhances viral replication, which is reduced following successful TB treatment and resolution of the TB-associated proinflammatory microenvironment. As such, HIV-1 VL has been demonstrated to significantly correlate with *Mtb*-induced plasma and pleural fluid cytokine levels in TB patients [31–33].

In a case–control study of persons living with HIV-1 with similar levels of immune suppression (absolute CD4 counts), TB cases had a higher incidence of other new AIDS-defining opportunistic infections (OI) and reduced survival, supporting the case that TB potentiates AIDS progression and HIV-1 induced morbidity and mortality [34]. As an AIDS defining illness in persons living with HIV, TB is unique compared with other OI in that the increased risk for TB can occur before significant CD4 depletion [31] and at a log lower HIV-1 VL than other OI [28,35]. This suggests that *Mtb* infection imparts a broader dysfunction on immune cells in HIV-1, than merely CD4 depletion, leading to much earlier HIV-1 disease progression than other opportunistic pathogens. While the contribution of TB disease to HIV-1 progression is unrefuted, the contribution of LTBI to AIDS development has been little explored in the literature, based solely on plasma VL and CD4 changes [36]. Recent studies investigating *Mtb*-induced immune activation and changes in the inflammatory mileu at infected sites potentially provide support for a larger contribution of LTBI to HIV-1 progression than is currently acknowledged [37].

The dramatic increase in incidence of TB and TB-associated mortality following the expansion of the HIV-1 epidemic has shifted the focus of the research and medical community to understanding and intervening in HIV-associated TB progression. HIV-1 coinfection complicates and worsens TB outcomes. The early development of TB in HIV-1-infected individuals has been linked to a propensity of HIV-1 to infect activated *Mtb*-specific T-cells, leading to preferential depletion and alteration of the phenotype and function of TB adaptive immunity in the lungs [38–42]. This early T-cell depletion has been suggested to cause TB granuloma disorganization, loss of *Mtb* containment and thus increased *Mtb* dissemination, resulting in higher prevalence of extrapulmonary TB [43]. In some respects, HIV-1 causes a disease more similar to primary progression in infants [44]. T-cell depletion also decreases sensitivity to diagnose *Mtb* sensitization, based on antigen-specific T-cell assays [45]. The exacerbation of TB severity and mortality by HIV-1, concomitantly reducing TB transmission is, however, counterintuitive to a syndemic benefit between these pathogens. HIV-1 in effect causes a ‘dead end’ for the bacilli. Conversely, if *Mtb* infection increases HIV-1 replication, and thus risk of transmission, is *Mtb* rather a greater benefit to the HIV-1 epidemic than HIV-1 is to *Mtb*?

## Acquisition of infection

### Latent *Mtb* infection

LTBI represents the circumstances where individuals maintain *Mtb* infection by a delicately controlled innate and adaptive immune cell interaction, controlling *Mtb* replication, but preventing immune exacerbation [46,47]. Latently infected individuals represent the largest reservoir for potential reactivation and transmission. Inhaled *Mtb* bacilli are encapsulated by a collection of immunological cells, including infected and recruited alveolar macrophages, neutrophils, differentiated monocyte-derived macrophages (MDM), epithelioid cells and multinucleated giant cells, Langhans giant cells, surrounded by T and B lymphocytes and other non-classical innate cells, which organize themselves into granulomas [48].

**Figure 3.**
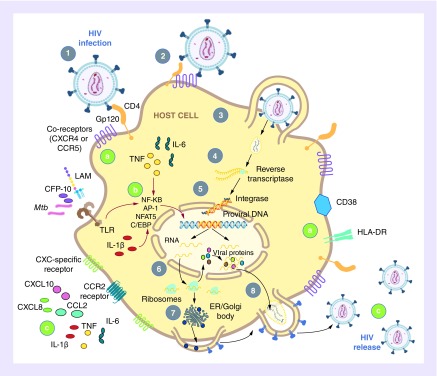
HIV-1 lifecycle and points where *Mycobacterium tuberculosis* coinfection may influence this lifecycle. **(1)** Viral attachment requires high affinity binding of the HIV-1 Envelope Gp120 to the host cell CD4 receptor. **(2)** HIV-1 interacts with either coreceptor CCR5 or CXCR4, forming a heterodimer with CD4, facilitating viral entry. **(A)** High levels of CCR5/CXCR4 coreceptors correlate with TB disease and activation markers CD38 and HLA-DR. **(3)** The Envelope Gp41 subunit protein then facilitates viral fusion and injection into the target cell membrane. **(4 & 5)** The viral enzyme Reverse Transcriptase converts RNA to cDNA and ultimately double-stranded DNA is produced, which is translocated to the nucleus, where the virally encoded integrase facilitates viral DNA integration into the host genome. **(6 & 7)** Once integrated, the virus utilizes host cellular machinery and energy to transcribe viral mRNA for packaging into new viral particles and for viral protein production. **(B)** HIV-1 replication may be enhanced by the proinflammatory environment created by *Mtb* infection or *Mtb* product (e.g., secreted CFP-10 or cell wall LAM) triggering of TLRs. Proinflammatory cytokines such as TNF, IL-6 and IL-1β induce transcription factors (NF-κB, AP-1, NFAT5, C/EBP), which readily bind to the viral LTR, and upregulate the transcriptional capacity of the virus. **(8)** The viral protease enzyme cleaves polypeptides needed for new virion assembly, which bud off the cell membrane through exosome transport. **(C)** *Mtb* may promote the establishment of a larger pool of cell-free viral particles and increase recruitment and activation of infection-susceptible cells through increased chemokine production, such as CXCL10, CXCL8 and CCL2 and proinflammatory cytokine secretion. LAM: Lipoarabinomannan; LTR: Long terminal repeat; *Mtb: Mycobacterium tuberculosis*; TB: Tuberculosis; TLR: Toll-like receptor.

Formation of the *Mtb* granuloma during latent infection is associated with a strong localized and systemic proinflammatory response. Bacterial activation of surface toll-like receptors on phagocytes induces TNF, IFN-γ, IL-1β, IL-6, IL-12, IL-10 and TGF-β, activating phagocyte and recruited T-cell functions (Figure 3) [46,47]. A major systemic component in serum before disease onset are proteins CXCL9-10, CCL5, CCL2 and CXCL8, predominantly recruiting T-cells, monocytes and neutrophils, respectively, to the site of *Mtb* infection [49].

Granulomas benefit the host by containing and restricting mycobacteria, ultimately preventing dissemination. Studies using transparent zebrafish infected with *Mycobacterium marinum*, to model early stages of mycobacterial infection, showed the formation of epithelioid granulomas prior to activation of the adaptive immune response, coinciding with wide bacterial expansion [50]. These findings suggest that granuloma formation may also be used by bacteria to increase infection and dissemination. T and B cells recruited to help form the granuloma are primed in the lymph nodes (LN) by migrating dendritic cells (DCs) presenting *Mtb* antigen [51,52]. However, it has also been suggested that *Mtb*-infected neutrophils and DC will traffic live *Mtb* to LN, setting up the LN as a site of *Mtb* reservoir during latency [53,54]. Live *Mtb* bacilli have been isolated from lung granulomas of individuals with clinically inactive TB, demonstrating the ability of viable organism to persist during latent infection [55] and multiscale modeling predicts *Mtb* populations are highly dynamic during latent infection responding to the host inflammatory environment, countering the idea that all latent *Mtb* resides in a dormant nonreplicating state [56,57].

### HIV-1 acquisition

#### Crossing the primary barrier

HIV-1 is transmitted via infected bodily fluids, including seminal and vaginal fluids during sexual intercourse; blood during transfusions or blood contact between broken skin and wounds; intravenous drug use and from mother to child (*in utero*, during delivery or through breastfeeding). HIV-1 can cross the outer epithelial lining of skin tissue, which acts as the primary physical barrier to infection. Parts of the body covered by mucosal membranes, ‘wet skin’, including genital and rectal tissues, are particularly vulnerable to HIV-1 infection and are primary sites of mucosal infection. HIV-1 crosses the mucosal epithelial barrier through capture by DCs, transcytosis through the intraepithelial layer, attachment to Langerhans cells and CD4^+^ T-cells and/or the disruption of the mucosal membrane integrity by HIV-1 envelope glycoprotein 120 (Gp120) (reviewed in [58]).

#### Viral entry into cells

Viral attachment requires high affinity binding of HIV-1 Gp120 to the host CD4 receptor. HIV-1 interacts with either coreceptor C-C chemokine receptor 5 (CCR5) or C-X-C chemokine receptor 4 (CXCR4), forming a heterodimer with CD4, facilitating viral entry (Figure 3). The HIV-1 envelope Gp41 subunit protein then facilitates viral fusion and injects itself into the target cell membrane. Studies show that mutated coreceptors and/or coreceptor inhibitors lead to reduced HIV-1 infectivity [59–61]. Strains which predominantly infect using CCR5 are referred to as R5-tropic, strains which use CXCR4 are called X4-tropic, and dual-tropic R5/X4 strains infect cells expressing either coreceptor [62]. Transmitted viral strains are predominantly R5-tropic while X4 strains are seldom observed in early infection [63]. Within an infected individual, the viral population may shift from R5 to X4 over time, as it evolves in the natural course of infection, and this switch correlates with subsequent rapid disease progression [62].

Frontline mucosal-resident T lymphocytes, tissue macrophages and DCs are primary targets for HIV-1 infection. Of these, primary mucosal CD4^+^ T lymphocytes are the predominant target, due to their high surface expression of CD4, showing increased susceptibility to infection *in vitro* as well as increased levels of viral replication. Increased surface expression of CCR5 and T-cell activation status also contribute to the preferential infection of these cells [64]. HIV-1 infects monocytes and macrophages due to their high CCR5 and moderate CD4 surface expression [64,65]. HIV-1-infected tissue-resident and MDM can fuse with CD4^+^ T-cells enabling direct viral transmission between these cells [66].

Following HIV-1 entry into host cells, the viral reverse transcriptase enzyme converts RNA to cDNA and ultimately double-stranded DNA, which is translocated to the nucleus, where the virally encoded integrase facilitates viral DNA integration into the host genome [67]. Once integrated, the virus uses the host cellular machinery and energy to transcribe viral mRNA for packaging into new viral particles and for synthesis of its protein components. The viral protease enzyme cleaves polypeptides needed for assembly of new virions, which bud off the cell membrane through exosome transport (Figure 3, reviewed in [68]).

Several host chemokines that are natural agonists of CCR5 may act as HIV-1 antagonists during HIV-1 infection, by blocking Gp120 interaction with CCR5. C-C chemokines CCL3 (MIP1α), CCL4 (MIP1β) and CCL5 elicit a dose-dependent inhibition of HIV-1 Gag p24 release from peripheral blood mononuclear cells (PBMCs) [69]. These chemokines specifically inhibit the infection of R5-tropic HIV-1 strains, suggesting that the mechanism of action is via impeded viral entry [70]. Paradoxically, these chemokines increase replication of X4-tropic viruses. Experiments with PBMC, depleted of CD8^+^ T-cells and infected with a X4 HIV-1 strain, in the presence of supplemented CCL5, exhibited an increased cellular reverse transcriptase activity, compared with untreated PBMC. This suggests that CCL5 may induce viral replication in X4-infected cells [71]. Patients with high plasma CCL5 concentration also show higher levels of plasma X4 HIV-1 RNA copies [72]. These observations suggest that C-C chemokines may be important early in blocking R5 HIV-1 infection, but detrimental to the host as the viral infection progresses and more X4-tropic viruses emerge.

#### Establishing HIV-1 infection

Following transmission, the founding HIV-1 variant/s rapidly expand and spread beyond the site of infection to other parts of the body via the bloodstream and lymphatic vessels that lie within the mucous membrane tissue. Rapid homing of the virus to the gut-associated lymphoid tissue, facilitated by the integrin α4β7 gut homing receptor has been demonstrated to play a major role in the early expansion of HIV-1 within an infected individual as this anatomical site harbors high levels of CD4^+^CCR5^+^ T-cells (reviewed in [73]). A larger, self-propagating viral pool is then established and can cause systemic infection in secondary lymphoid tissue sites [74].

An ‘eclipse phase’, a period of approximately 10 days during which viral RNA is undetectable in the plasma, precedes the detection of viral antigen [75,76]. Peak viremia is reached at approximately 2 weeks postinfection [77], followed by significant VL reduction to a resting steady state, otherwise referred to as the viral ‘set point,’ and the onset of the chronic phase of infection (Figure 2) [78]. This set point may vary up to 1000-fold between patients, with a higher set point 6 months after seroconversion as well as the decline in CD4 counts being early predictors of rate of disease [79–81]. However, a change in VL is less predictive in the years preceding AIDS onset, while the pattern of CD4 decline is predictive [82]. Thus, a higher VL immediately following infection and greater declines on CD4 T-cells are important defining features of the severity of outcome of infection [83].

#### Enhancers of HIV-1 acquisition, expansion & replication

Multiple factors have been characterized that enhance the ability of HIV-1 to both establish infection and replicate within the infected host cell. Increased inflammatory cytokine levels at the genital mucosa, potentially linked to an existing sexually transmitted infection, have been associated with increased risk of HIV-1 acquisition [84] and expanding the transmission bottleneck [85]. These cytokines facilitate recruitment and activation of cells targeted by HIV-1. Inflammation at this site has also been associated with creating an environment that is more permissive to infection by less infectious viral variants [86].

The mode of infection has also been linked to the rate of HIV-1 expansion at the site of infection and dissemination into secondary lymphoid tissues. Cell-to-cell transmission, facilitated through viral synapses, is proven to be a significantly more effective means of viral expansion than cell-free virus-mediated spread [87]. This is the case for spread of HIV-1 from T lymphocytes to MDM, and from infected macrophages to other permissive cells [88]. Migration of HIV-1-infected antigen-presenting cells (APCs) particularly to the LN and associated tissue increases the chance of infecting resident macrophages and T lymphocytes, thus further spreading HIV-1 infection [89]. Receptor tropism is also associated with expansion of HIV-1 within a host whereby X4-tropic viruses, more prevalent in late stages of infection, expand more rapidly as they infect a broader range of cells and have enhanced pathogenicity [90,91].

Within cells, HIV-1 replication is enhanced by the presence of proinflammatory cytokines such as TNF, IL-1β and IL-6 [92,93], which induce human transcriptions factors (hTFs) that bind the viral promoter, the long terminal repeat (LTR), upregulating the transcriptional capacity of the virus [94,95]. The LTR has binding motifs for hTFs including NF-κB [94], AP-1 [96], SP1 [97], C/EBP [98] and NFAT5 [99]. Coinfections that create proinflammatory environments conducive to LTR activation will benefit HIV-1 by favoring viral replication, at the site of acquisition or secondary lymphoid reservoir sites (Figure 3).

#### Mtb infection creates an expanded cellular niche susceptible to HIV-1 infection

*In vitro* infection of CD8^+^ T-cell-depleted LN lymphocytes and PBMC isolated from HIV-1-infected individuals with and without LTBI, indicated HIV-1 replication was only induced in cells isolated from those with LTBI, hypothesized as a consequence of the preferential infection of activated *Mtb* antigen-specific T-cells by HIV-1 [29]. *Mtb* infection may favor HIV-1 infection by increasing the density of viral entry coreceptors, CCR5 and CXCR4, on the surface of CD4^+^ *Mtb* antigen-specific T-cells and various myeloid populations [100–102]. The recruitment of such T-cells to the site of HIV-1 acquisition would result in the establishment of a larger pool of virus, expanded viral reservoirs and consequently, a higher viral set point during the acute phase of infection. As HIV-1 is not incredibly efficient at establishing infection, creating an environment with a diversity of cells with increased viral receptor density would benefit the virus [103].

Clinical evidence to support the early recruitment of circulating *Mtb*-specific T-cells to genital mucosa is difficult to obtain, although their circulating abundance and higher chemokine receptor density would support this likelihood. Clearly, their peripheral circulation would have direct relevance to increased HIV-1 acquisition following intravenous exposure.

Increased density of HIV-1 coreceptors on *Mtb*-specific T-cells is not the sole mechanism by which LTBI may enhance HIV-1 acquisition and established infection. Blood from pulmonary TB patients had significantly increased CCR5^+^ T-cells, as well as increased CXCR4^+^ and CCR5^+^ monocytes [100]. T-cells isolated from PBMC stimulated with mycobacterial species *M. bovis* BCG and *Mtb* are also more susceptible to R5 and X4 HIV-1 infection, compared with unstimulated T-cells or cells infected with *M. smegmatis*. This effect is abrogated by TLR2 silencing in PBMC, supporting the concept that monocyte/macrophage proinflammatory cytokine production during *Mtb* infection enhances bystander T-cell HIV-1 susceptibility [104]. *Mtb*-specific CD4 T-cells, CD27^+^CD57^-^, also show increased susceptibility to HIV-1 infection, correlating with reduced CCL4 secretion, hypothesized to facilitate greater CCR5–Gp120 interaction [105].

Early studies of newly diagnosed HIV-1 and TB-HIV-1 patients indicated that HIV-TB exacerbates immune activation, with elevated HLA-DR expression on CD4^+^ and CD8^+^ T-cells, correlating with elevated expression of Fc receptors I and III on peripheral monocytes [106]. Recently, in whole blood transcriptomic analyses, elevated Fc Gamma Receptor signaling and classic complement activation were the greatest predictors of subclinical TB infection and risk of TB progression in ART naive HIV-1-infected individuals with LTBI [107]. More complex multiparameter flow cytometry of antigen-specific T-cells also indicated that TB-HIV-1 patients, compared with TB only, have a higher frequency of IFN-γ producing *Mtb*-specific CD4^+^ and CD8^+^ T-cells with elevated activation markers HLA-DR and CD38 and higher TNF production. By contrast, IFN-γ producing CMV-specific T-cells show no elevated activation. This indicated that immune activation in HIV-1 is specific to *Mtb* antigen-specific T-cells, and not nonspecific activation of any antigen-specific T-cell [108,109]. These findings support the hypothesis that *Mtb* infection creates a larger circulating pool of innate and adaptive immune cells susceptible to HIV-1 infection, which can increase both risk of HIV-1 infection, quicker expansion of infection during acute infection and thus enhanced HIV-1 progression during chronic infection.

#### Mtb impact on HIV-1 viral replication & set point

LTBI may increase HIV-1 viral set point, leading to an increase in the severity of HIV-1 disease in TB endemic regions [80]. This hypothesis is supported by multiple lines of evidence including that infection with *Mtb* increases HIV-1 risk of infection [101,104] and the rate of replication both *in vivo* [31,110] and *in vitro* [21,22]. Mathematical modeling predicts that coinfected patients have a VL set point 27% greater than HIV-only infected patients [111]. With the support of their model, the authors argue that a high VL set point increases the risk and timing of coinfected persons progressing to AIDS, by 2 years.

It has long been known that coinfection of the same cell by *Mtb is* not necessary to induce HIV-1 replication in PBMC [112]. In 1997, Nakata *et al.* investigated the *in vivo* effect of coinfection with *Mtb* using bronchoalveolar lavage (BAL) fluid from HIV-1-coinfected TB patients compared with individuals with no lung disease, demonstrating a striking increase in HIV-1 VL in BAL from *Mtb*-infected lung segments. In each patient tested, the amount of HIV-1 RNA was greater in the infected lung segment versus the uninfected segment, suggesting that local inflammation increased HIV-1 replication and VL. BAL fluid levels of HIV-1 were also increased in all patients compared with plasma levels [113]. In 2001, Toossi *et al.* first showed increased HIV-1 replication at sites of active *Mtb* infection in pleural fluid from pleural TB patients. Increased replication was associated with increased *TNF* and *CCL2* expression, decreased amounts of HIV-1 entry blocking C-C chemokines (CCL3, CCL4, CCL5) and upregulation of CCR5 on pleural fluid mononuclear cells [114]. Such a suppression of CCR5 ligands, with concomitant CCR5 increase, would increase the propensity for R5-ropic virus infection. While these observations were made in fluid isolated from TB patients, the impact on HIV-1 at the local site of *Mtb* infection, supports the hypothesis that such a relationship would occur in any tissue site where HIV-1 and replicating *Mtb* coexist, prior to the development of symptomatic TB disease.

Later studies implicated the proinflammatory cytokine milieu in enhancing replication of HIV-1 through hTF activation of the LTR [115]. Alveolar macrophages isolated from TB patient BAL, compared with noninflamed lungs from healthy controls, have downregulated expression of the inhibitory C/EBPβ isoform and increase expression of the C/EBP-activating isoform, facilitating increased HIV-1 replication [116]. In an *in vitro* PBMC coinfection model, HIV-1 replication had a magnitude-dependent cytokine-induced increase, relative to the strain of infecting *Mtb* [22]. Coinfection with the proinflammatory *Mtb* Lineage 4 strain CDC1551 significantly increased supernatant HIV-1 Gag p24 protein concentrations, compared with infection with Lineage 2 HN878, a less inflammatory strain which produces the cell wall phenolic glycopid [22]. This strain difference was abrogated by deletion of phenolic glycopid from HN878 [22] or by silencing the expression of *NFAT5* induced by CDC1551 [21]. Silencing of the TLR downstream adapters, MyD88, IRAK1 and TRAF6, in human monocytes also inhibited *Mtb*-induced *NFAT5* expression and HIV-1 replication during CDC1551 coinfection [21]. ZNF134 has recently been identified as a novel HIV-1 LTR activator, which is induced to a greater extent during mycobacterial infection compared with HIV-1, and PBMC from TB patients demonstrate high levels of *ZNF134* expression, even in the absence of HIV-1 [117]. These studies demonstrate that *Mtb* cell wall components activate hTFs via TLR signalling, inducing HIV-1 replication. Importantly, *Mtb* culture filtrate proteins or purified cell wall mannose-capped LAM can independently activate HIV-1 LTR transcription, in the absence of live bacilli. This suggests bacterial products within the granuloma milieu could activate HIV-1 transcription in bystander *Mtb*-uninfected cells [118]. Coinfection within the same cell is thus neither required, nor likely the predominant effect via which *Mtb* affects HIV-1 replication and infection within the same individual.

In further support of this, coinfection with *Mtb* may also contribute to elevated levels of systemic immune activation, which will potentiate HIV-1 disease pathogenesis. Macrophage and T-cell-specific soluble cellular markers of immune activation, sCD27, sCD163, IL1RA and sCD14, are found at increased levels in pleural fluid versus plasma, and even higher in TB patients coinfected with HIV-1 [119]. Soluble monocyte activation markers, sCD14, IL-6, CXCL10 and CRP, are elevated in coinfected patients with active but not latent infection. However, the same HIV-1-infected individuals were found to have elevated lymphocyte activation with higher surface expression of CD38 and HLA-DR on CD4^+^ and CD8^+^T-cells when latently *Mtb* infected, increasing the propensity of HIV-1 for T-cell infection [37].

### HIV-1 immune evasion & viral spread

#### Cytolytic T-cell responses & ADCC

The success of HIV-1 infection is governed by early immune evasion and ineffective viral clearance. Natural killer (NK) cell and HIV-specific CD8^+^ T-cell responses arise shortly after infection and are pivotal in the outcome of infection [120]. CD8^+^ T-cell responses have been proposed to play a role in reducing peak viremia to a stable state set point VL [121]. However, due to the error prone HIV-1 reverse transcriptase that misincorporates nucleotide bases into the viral genome [122], these responses are rapidly (within weeks) escaped through selection of amino acid mutations in HLA-presented epitopes [123]. Likewise, evidence of escape from NK cell responses have been reported [124]. Antibody responses confer even less benefit to control of HIV-1. Those that are able to neutralize the virus arise somewhat later in infection (months) [125,126] and are likewise rapidly escaped through mutations in the viral envelope.

Exploitation and evasion of these immune-targeted responses enables the virus to replicate and persist despite the host’s best efforts to eradicate HIV-1. Viral accessory proteins, Vif, Nef, Vpu and Vpr, modify the hostile host cell environment and facilitate viral escape from cell-mediated and innate immune responses, through a variety of mechanisms (reviewed in [127]). Viral protein-induced CD4^+^ T-cell apoptosis and cell death of bystander cells, potentially induced by both host factors (e.g., TNF, Fas ligand and TRAIL) and various HIV-1 factors (Tat, Vpr and Nef), disable CD4^+^ T-cell secretion of inflammatory cytokines and impact their action on APC [128,129].

Antibody-dependent cellular cytotoxicity (ADCC) is a highly effective host control mechanism mediated predominately by NK cell activation of cytolytic T lymphocytes (CTLs). The intricate activating and inhibiting receptor combination presented by NK subsets facilitates a highly coordinated response to sensed ligands and CTL activation. Nef proteins downregulate surface expression of the NK ADDC-activating receptor NKG2D, reducing ADCC susceptibility [130]. Similarly, HIV-1 proteins reduce surface expression of CD4 and presentation of CD4-Env epitopes, to evade ADCC [131,132].

HIV-1 has also been shown to modulate the expression of MHC Class I and II proteins [133]. HIV-1 infected cells can avoid the CTL response and subsequent killing by Nef-mediated downregulation of surface MHC I [134]. MHC II restricted peptide presentation to specific T-cells is inhibited by Nef, reducing the activity of CD4^+^ T-helper cells required for control of viral infection [133].

#### Unclear role of latent Mtb in HIV-targeted ADCC

While CD8 and NK cells play an important role in ADCC and CTL-mediated killing of HIV-1-infected cells, their role in *Mtb* control is far less defined or as studied as CD4^+^ T-cells. NK cells are found in human TB granulomas and their ability to produce IFN-γ and TNF during *in vitro Mtb* infection varies by blood donor KIR genotype [135]. NK cells from TB patients during disease have reduced IFN-γ and degranulation, compared with post-treatment, suggesting *Mtb* infection that progresses to disease impairs NK function [136]; this would benefit HIV-1 escape from CTL killing. As macrophage cross-priming via MHC-I optimizes CTL responses, *Mtb*-induced impairment of cross-presentation [137] may further contribute to CTL evasion.

A recent study identified distinct PPD-specific Fc Gamma antibody profiles between latent *Mtb* infected and active TB patients [138], yet there were no uninfected controls (controlling for other mycobacteria) or post-treatment responses to determine whether the difference arose during TB disease or in those at risk of developing the disease when *Mtb* infected. Antibody differences were associated with PPD-specific reduced NK cell-mediated ADCC, degranulation (CD107a) and CCL4 and IFN-γ secretion in those with active TB [138]. How *Mtb* infection may truly impair ADCC and CTL-mediated HIV-1 killing *in vivo* during LTBI is far from elucidated and requires greater investigation.

### Viral reservoirs & latency

#### Cellular targets of latent HIV-1

Despite the unquestionable success of ART to reduce viral replication and improve the quality of life of HIV-infected people, HIV-1 continues to persist latently within infected host cells and tissues, in the absence of viral replication [139–142]. This may be caused by unsuppressed low levels of HIV-1 replication within drug-privileged anatomical sites, but has predominantly been characterized in cells harboring integrated, transcriptionally silent viral genomes. The large majority of these integrated viral genomes are defective and harbor internal deletions or are hypermutated [143,144]. These persistent quiescent cells can escape host-targeted killing by immune responses and drug targeting, as current drug regimens target stages of the life cycle prior to or postviral integration. Quiescent-infected cells can re-enter a productive HIV-1 life cycle in response to various stimuli, including antigen-specific T-cell activation and inflammatory cytokine induction in the tissue microenvironment (reviewed in [142]).

A pool of latently HIV-1-infected cells is established during the early stages of HIV-1 infection, when infected patients are untreated. Despite initiation of ART as early as 3 days postinfection, this pool is established [145] and is highly stable and long-lived [140,146], owing largely to maintenance through homeostatic proliferation [147]. These cells may originate from infected and activated HIV-1 antigen-specific cells that differentiate into long-lived resting memory T-cells or may be directly established in inactivated resting CD4^+^ T-cells. As HIV-1 exhibits a narrow tropism for CD4^+^ T-cells, this pool of cells represents a large, if not the largest, viral reservoir in an infected host [139,148]. Two different sets of resting CD4^+^ T-cells exist: the naive and the memory CD4^+^ T-cells. Naive cells can carry HIV-1 DNA and replication competent HIV; however, their frequency of infection is usually much lower than that of the memory CD4^+^ T-cells [142].

There is mounting evidence that other nonconventional viral reservoirs exist within the host (reviewed in [149–151]). Tissue macrophages and/or monocytes enable viral persistence by harboring viral particles and relocating them to inaccessible immune-privileged sites, where they can evade the host and ART restriction. At the same time, macrophage functioning is impaired by HIV-1 proteins (eg. Nef, Gag, Tat and Env) permitting a unique environment for HIV-1 to persist [152]. Unlike the apoptotic cascade induced in activated CD4^+^ T-cells; in macrophages, HIV-1 blocks apoptosis, prolonging survival, through degradation of the host long noncoding RNA lincRNA-p21. This prevents activation of its apoptotic partner hnRNP-K, removing lincRNA-p21 inhibition of the prosurvival MAP2K1 [153]. It is estimated that HIV-1 proviral DNA is present in less than 1% of monocytes [154]. Other studies indicate approximately 50 per 10^6^ LN macrophages are infected [155]. Infectious HIV-1 has also been detected in circulating monocytes from patients initiated on ART for extended periods. Importantly, undetectable amounts of HIV-1 RNA are produced under basal conditions, but viral reactivation can occur following coinfection with opportunistic infections [156].

#### Tissue targets of latent HIV-1

Several organs serve as viral tissue reservoirs that enable the persistence of HIV-1 infection in the presence of ART [148]. The LN and lymphoid tissues are major tissue reservoirs of HIV-1, enabling viral replication, production and persistence and the storage of viral particles within immune complexes [157,158]. Replication-competent virus has been recovered in cells isolated from LN of virally suppressed patients. Both naive and central memory T-cells (TCM) are selectively retained in the LN, due to their homing receptor expression and DCs also accumulate in the LN, all with the potential to aid in viral expansion (reviewed in [159]). Central memory peripheral T follicular helper (Tfh) cells, in particular those PD1^+^CXCR3^-^, were identified as the major circulating T-cell reservoir, also being highly susceptible to HIV-1 infection. Frequencies of activated HLADR^+^ CD38^+^ CD4^+^ T-cells correlate with the level of viral induction in peripheral Tfh cells, suggesting systemic immune activation contributes to reservoir maintenance and functionality [158].

Other lymphoid organs such as the spleen, thymus and bone marrow have also been implicated as potential HIV-1 reservoirs (reviewed in [160]). Bone marrow, as a secondary lymphoid organ and site of haematopoiesis, contains progenitor cells that are not only long-lived, but divide and thus can act as replicating nodes, producing progeny with integrated HIV-1 DNA. In ART-naive individuals, HIV-1 has been identified in bone marrow CD133^+^ hematopoetic stem cells, mesenchymal stem cells, macrophages and memory CD4^+^ T-cells. CD4^+^ T-cells expressing the surface receptor CCR6, a marker of Th17 cells with homing capacity to the gut, are highly sensitive to HIV-1 infection. This also implicates the gut as a potential HIV-1 reservoir [161,162].

The lungs are very active immunological effector sites constantly being exposed to viral and bacterial airborne pathogens and represent potential HIV-1 lymphoid reservoirs. The lungs have features that render them ideal ‘sanctuaries’ for HIV-1 persistence. Cell-to-cell viral spread may be promoted by the proximity of millions of alveoli that provide a large surface area for persistence and an abundance of macrophages and DCs. Alveolar macrophages that are abundant in the lung spaces provide an ideal niche for persistent HIV-1 due to their resistance to HIV-induced apoptosis [163]. *Mtb* has also been shown to increase the surface expression of CXCR4 on alveolar macrophages, thus enhancing their susceptibility to X4 HIV-1 [101].

Differing levels of HIV-1 within the lungs, in comparison to peripheral blood, have been documented. In ART naive individuals HIV-1 has successfully been isolated from lung cell-free BAL fluid, CD4^+^ and CD8^+^ T-cells and alveolar macrophages [150,164]. Alveolar macrophage infection is associated with weakened phagocytic functioning; and HIV-1 RNA and DNA have also been detected in these macrophages from ART-initiated patients [165,166]. During ART, macrophages within sanctuary sites may, therefore, be a source of persistent HIV-1 viremia, although their role here remains controversial [167].

#### Mtb contribution to HIV-1 reservoir abundance & activation

Pulmonary TB patients have increased CCR5^+^ CD4^+^ T-cells in the BAL fluid and lower respiratory tract when compared with healthy controls or non-TB lung disease controls [102]. Comparing blood and BAL cellular compartmentalization in *Mtb* latently infected individuals indicates those that are HIV-1 infected have higher numbers of CD4^+^ T-cells in the lung, despite moderate peripheral reduction in CD4^+^ T-cells. There was a positive correlation with absolute number of CD4^+^ and CD8^+^ T-cells in the BAL and BAL VL, while an inverse relationship was found in the periphery with plasma VL and CD4^+^ T-cells. Three quarters of BAL CD4^+^ T-cells were positive for CCR5 and thus potential targets of HIV-1 infection [42].

Given the efficiency of direct cell-to-cell viral transmission [88], the TB granuloma provides an ideal permissive environment for HIV-1 infection and spread due to its tightly packed cellular architecture. *Mtb* has been shown to exacerbate HIV-1 infection by increasing viral transfer between cells, using tunneling nanotubes formed from IL-10-stimulated macrophages [168]. HIV-1 infection of LN-migrating *Mtb*-infected macrophages and DCs also has the potential to enhance the lymphatic spread of HIV-1 during ART [169]. It could be hypothesized that *Mtb* infection stimulates trafficking of HIV-1-infected peripheral circulating monocytes or HIV-infected CD4^+^ T-cells from the LN to the site of *Mtb* infection, thereby providing quiescent HIV-1-infected cells new tissue-resident target cells, and exposure to a proinflammatory tissue environment, to reactivate viral replication. Alternatively, trafficking *Mtb*-infected cells may coinfect a HIV-1-infected lymphatic microenvironment, resulting in recruitment of activated peripheral monocytes and T-cells to the LN, delivering a pool of HIV-1 susceptible cells to the HIV-1-infected environment (Table 1). These potential kinetic movements need *in vivo* validation.

During latent infection, *Mtb* has been identified in circulating human CD34^+^ peripheral hematopoetic stem cells and CD127^+^ bone marrow mesenchymal stem cells [170], suggesting that bone marrow may also be a reservoir for *Mtb* [171] as well as HIV-1. Enhanced recruitment of *Mtb*-specific T-cells to the bone marrow would create another niche for viral expansion and replication. During early ART reconstitution, the strongest correlate of restored *Mtb* immunity is an expanded CD27^+^ TCM response, the predominant T-cell reservoir for HIV-1 [172]. Thus, the coexistence of latent *Mtb* and HIV-1 reservoirs in the same tissue microenvironments has the potential for expanded HIV-1 infection and cell-to-cell spread during ART.

Extrapulmonary TB is much more common in HIV-1-infected individuals, and this occurs at sites where HIV-1 reservoirs are predominately found, particularly, LN, brain and cerebrospinal fluid, kidneys, liver, spleen and bone marrow, whilst some HIV-1 lymphoid reservoirs (thymus, gut-associated lymphoid tissue) are less common sites of TB (reviewed in [173,174]). With the exception of T-cell populations in the LN; macrophages, DC, astrocytes, microglia and epithelial cells are the commonly identified HIV-1 reservoir cells in these tissues and autopsy studies demonstrate *Mtb* DNA presence in these cells in latent infection [171,175]. The order of pathogen arrival in these sites is unclear and likely to exist in both directions, in any one individual.

Inflammatory DCs and MDM are capable of carrying *Mtb* from the granuloma to LN [176,177]. Neutrophils are also able to participate by harboring and transporting viable *Mtb*, which can be subsequently phagocytosed by tissue macrophages after transport [178]. Pre-existence of *Mtb* in these lymphoid sites would have the propensity to favor viral expansion once HIV-1 arrives in the LN.

Conversely, CD16^+^ monocytes that are found in greater frequency in *Mtb* infection [179] are susceptible to HIV-1 infection [180] and show increased transmigration from bone marrow, especially to the CNS, during HIV-1 infection [181]. Activated monocytes could, therefore, traffic HIV-1 to peripheral *Mtb*-infected sites, along a CCL2 gradient, thus setting up a peripheral site of coinfection. We postulate that pre-existence of *Mtb* in tissues that are common HIV-1 sanctuaries, as well as increased transmigration of HIV-infected APC to sites of LTBI both contribute to the higher frequency of extrapulmonary TB progression in HIV-infected individuals.

### Defining coinfection dynamics in HIV/*Mtb* animal models

In the majority of clinical studies, determining the sequence of coinfection and dynamic changes during HIV-1 progression at diverse anatomical sites is difficult to determine, although critical to the understanding of the key effectors of *Mtb* infection on HIV-1 progression. Whether *Mtb* has a greater effect on HIV-1 acquisition risk or progression, and whether enhanced HIV-1 expansion is restricted to *Mtb* granulomas or extends to more diverse HIV-infected anatomical sites and tissue reservoirs, are key questions to inform any strategy designed to reduce the effect of *Mtb* on HIV-1 infection and progression.

Nonhuman primate (NHP) and bone marrow, liver and thymus (BLT)-humanized mice animal models allow researchers to build more detailed understanding of disease progression dynamics and the sequence of immune perturbations during coinfection, which clinical studies cannot answer. HIV-1 and *Mtb* coinfection models allow specific examination of pathogen strain, dose and duration, which reduces some variability observed in human studies. It also allows simultaneous investigation in plasma and tissues, including lung granulomas, LN, spleen and other extrapulmonary sites. Although no animal model completely recapitulates all aspects of any human disease, NHP studies of simian immunodeficiency virus (SIV) and *Mtb* closely resemble the full spectrum of both diseases (reviewed in [182,183]).

#### Variable impact of Mtb coinfection on SIV replication within plasma of coinfected NHP

NHP are unique in that they are one of the only animals who develop TB similar to humans, whilst also being a natural host of an immune deficiency virus (SIV) related to HIV. As an animal model of coinfection, they facilitate plasma sampling examined over an entire course of infection. SIV levels within plasma, over the course of disease, have been found to be similar within NHP with coinfected latent *Mtb* (CDC1551 or Erdman lab-adapted strains) infection compared with NHP infected with SIV alone [184–186]. There was no difference noted in peak viremia within coinfected and SIV-only infected macaques [185]. Interestingly, when SIV was used as a secondary infection to reactivate LTBI, no difference in SIV replication within the periphery was observed [186,187], suggesting that plasma viral set point did not change when TB was reactivated. Similarly, plasma SIV replication generally did not change within SIV-infected NHP from set point when *Mtb* infection followed SIV [188–190]. Taken together, these data suggest that *Mtb* and disease status do not change SIV replication within plasma. However, these observations counter human prospective HIV-TB cohorts [25] and it must be noted that lab-adapted SIV strains may not reflect naturally circulating strains or responses [191].

Studies examining infection with the live-attenuated TB vaccine *M. bovis* BCG and SIV coinfection found that NHP infected with BCG required fewer low-dose oral exposures of SIV to become SIV infected than their BCG naive counterparts [192], suggesting that BCG infection increased the number of SIV-susceptible cells and risk of SIV acquisition. BCG-infected NHP also developed a higher peak viral RNA count compared with uninfected NHP [192]. Similarly, SIV-infected NHP that were inoculated with BCG experienced a transient spike in plasma VL after 1–2 weeks [193]. Although these studies run counter to the *Mtb*/SIV coinfection studies, they demonstrate how mycobacteria have the capacity to increase SIV replication and set point and there may be significant interactions related to NHP and pathogen diversity in the models used [191,194].

#### Disease status & Mtb presence within tissue changes SIV or HIV-1 replication within coinfected animal models

One of the most significant benefits of HIV/*Mtb* coinfection animal models is the ability to examine viral replication and integration within a variety of *Mtb*-infected tissues. Within *Mtb* and SIV-coinfected NHP, SIV replication has been identified in lung granulomas [184–187] and thoracic LN [186]. This demonstrates that SIV replication ubiquitously occurs within a variety of tissues of coinfected NHP, but does not quantify how these tissues change SIV replication.

When quantifying the impact of change in VL during latent *Mtb* (CDC1551) progression to TB, a higher abundance of SIV RNA was observed within lung tissue of *Mtb*-infected NHP who progressed to TB disease following SIV infection, compared with coinfected NHP that did not reactivate following SIV infection [187]. Likewise, lung tissue from SIV/*Mtb*-coinfected NHP with active TB contained more SIV DNA than coinfected NHP with LTBI. Whether this relates only to tissue with granulomas is unclear from the published methodology [185]. This study also identified the colocalization of SIV RNA and *Mtb* bacilli within CD68^+^ macrophages, suggesting that macrophages might provide an opportunity for direct interaction between both pathogens. These data suggest that lung tissue from NHP with TB disease might behave as active reservoirs of SIV because they contain more CD4^+^ T-cells with integrated viral DNA and SIV replication and coinfectable macrophages than NHP with controlled LTBI. One potential difference between HIV-1 and SIV is the suggestion that macrophages are more readily infected with SIV and genetic differences between these two viruses may impact the relevance of macrophage SIV infection in NHP models to human macrophages [195].

NHP with active TB also contain more *Mtb* growth than ones with LTBI [196]. Investigating a correlation between *Mtb* and SIV abundance between disease states found no correlation between *Mtb* CFU growth and total SIV RNA [186] or SIV p28 protein [184] within the same lung tissue sections. Together, these studies provide a crude bulk level analysis, indicating at the tissue level, there is some evidence that inflammation during TB disease progression increases SIV replication and integration at the site of disease, while there is little direct correlation to pathogen abundance. These studies are far from conclusive, as they do not compare pathogen abundance at a single cell or single granuloma level. They also only quantitate live *Mtb* and not the presence of other bacterial products, nonreplicating *Mtb*, or the cytokine milieu induced at the local *Mtb* infection site and how these correlate with SIV infection and activation status.

BLT-humanized mice have also been used to study the interaction between HIV-1 and *Mtb in vivo*. A higher frequency of HIV-1 Gag p24:CD4^+^ T-cell ratio was observed within lung interstitial tissue compared with spleen within HIV/*Mtb*-coinfected BLT-humanized mice [197]. Although the cellular composition within lung and spleen differ, this study suggests that CD4^+^ T-cells within *Mtb* diseased lung tissue are more susceptible to HIV-1 than CD4^+^ T-cells within spleen. Similarly, HIV-1 Gag p24 abundance within lung tissue of *Mtb*-coinfected BLT mice demonstrated a weak but positive correlation between *Mtb* CFU growth [198]. These humanized mice studies provide further evidence that HIV-1 can preferentially replicate within *Mtb*-infected lung tissue and that there exists increased recruitment of T-cells that have a higher susceptibility of infection either before or after reaching the infected lung.

### Implications of latent *Mtb* treatment on HIV-1 prevention & cure strategies

Latent *Mtb* treatment is currently recommended for individuals who are frequently exposed to active TB disease, at high risk of progressing to active TB disease or immunocompromized (e.g., HIV-infected). In high TB burden countries, the WHO recommends HIV-1-infected individuals are given 6-9 months or continuous isoniazid prevention therapy (IPT) for LTBI, while initiating ART or if not yet received. However, only an estimated 49% of HIV-1-infected individuals were reported to have initiated TB preventative therapy in 2018 [4]. The first 12-month trial of IPT at ART initiation resulted in a median reduction in TB by 37% over 2 years, although the greatest reduction in risk occurred during the 12 months while receiving IPT; there was a gradual increase thereafter, rather than a rapid increase, with IPT still protective from TB at 2 years [199]. Shorter, 6 month courses are associated with faster loss of benefit [200]. The fast spike after only 6 months of IPT may reflect insufficient clearance of latent *Mtb*, and a continual contribution of *Mtb* to faster HIV-1 progression.

Although the WHO recommends short-course (6–9 months) IPT at ART initiation for people living in high TB transmission countries, as there is no evidence that repeat treatment offers further protection, there are no recommendations for repeat IPT in the WHO guidelines. This is despite the fact that repeat exposure to *Mtb* in high incidence settings is likely occurring. If concurrent *Mtb* infection, irrespective of TB progression, potentiates HIV-1 progression and risk of transmission through increasing VL, then repeat IPT or alternative shorter course prophylaxis could benefit those living with HIV-1 at risk of *Mtb* reexposure.

Perhaps of most importance to HIV-1 eradication, is if *Mtb* infection is shown to impact the abundance, activation status and microenvironment of HIV-1 reservoir cells, this may have significant implications for the design and efficacy of cure strategies for those on long-term ART. The leading cure approach is the ‘Shock and Kill’ technique aimed at activating all quiescent HIV-1-infected cells, in order to leverage CTL and ADCC-mediated killing (reviewed in [201]). Thus, the impact of *Mtb* infection of inhibiting CTL targeting of HIV-1 infection is a critical question requiring more research. The second option ‘Lock and Block’ is the opposite, aimed at preventing HIV-1 from ever being activated [202]. The potential impact of *Mtb* infection activating viral reservoirs, during this approach, is more evident and should be taken into consideration in future study designs for such HIV-1 cure strategies.

## Conclusion

We have considered findings from epidemiological studies of TB-HIV coevolution, human and animal *in vivo* and *in vitro* studies in order to systematically assess the potential impact of *Mtb* infection, prior to TB development, on the progression and risk of HIV-1 infection. We acknowledge that potential confounders may exist when characterizing direct links between *Mtb*-HIV-1 coinfection and HIV-1 disease progression. These include behavioral and socioeconomic drivers of infection with either pathogen in communities where prevalence and incidence rates of both are high. We have not exhaustively covered the effects of treatment for either pathogen nor the immune environment induced by infection with either pathogen that could play a role in disease exacerbation. Although animal studies are sparse, compared with clinical studies, they demonstrate that SIV and HIV-1 do infect *Mtb*-diseased tissue and exist in close proximity, increasing the probability that HIV-1 replication is directly affected in *Mtb*-infected human tissues.

The interaction between these pathogens is complex and interweaved. It is likely that in any one individual, all modes and dynamics of coinfection may exist at any one time. While the TB granuloma may be the site where an HIV-1-infected T-cell first encounters *Mtb*, the LN may be the site where an *Mtb*-infected DC first encounters HIV-1. Conversely, if *Mtb* exists in the LN prior to HIV-1 infection, then the LN may be the first site a migrating HIV-infected T-cell encounters *Mtb*. The likely impact of coinfection may not necessarily be reliant on the order of events, and who gets where first, but rather their interaction within a shared microenvironment once there, which both pathogens are levering to ensure their longevity and transmission to future hosts.

## Future perspective

The early era of HIV-TB research, focused on the role of *Mtb* coinfection on HIV-1 replication and immune cell susceptibility to infection. As we progress into an era of life-long ART, studies evaluating the impact of *Mtb* on the establishment, size and maintenance of the HIV-1 reservoir are of interest to determine the effect of LTBI on HIV-1 eradication. New multiomic single-cell technologies, including single-cell sequencing, RNA flow-FISH cytometry and whole tissue laser scanning microscopy could provide unprecedented sensitivity to assess the presence and metabolic state of both pathogens and the relative contribution of their spatial interaction within various microenvironments. Such studies using clinical samples could be augmented by animal models examining how *Mtb* changes HIV-1 replication in close proximity and if lung granulomas and peripheral sites of coinfection are HIV-1 reservoirs *in vivo*. Using a BLT mouse model, it is possible to block HIV-1 dissemination by limiting T-cell recirculation, through hindering the movement of migratory T-cells from LNs into efferent lymph [203]. Utilization of such *in vivo* manipulation of T-cell recruitment during LTBI could help dissect the contribution of recruitment to *Mtb*-infected sites and LN dissemination in HIV-1 progression and reservoir expansion.

Answers to the following questions will provide further insight into whether *Mtb* coinfection changes the dynamics of HIV-1 reservoirs and decreases the barrier to success of HIV-1 cure strategies:

- Does *Mtb* infection increase the establishment and size of the HIV-1 reservoir: the abundance and phenotype of infected cells and increased locations?
- Does *Mtb* infection, prior to HIV-1, favor early HIV-1 expansion to *Mtb*-infected LN and lung sites?
- Does HIV-1 replication increase in *Mtb*-infected granulomas and does replication correlate with the phenotype of infected cells or the cytokine environment in the granuloma?
- What role does *Mtb* infection have on gut HIV-1 infection and homeostasis, including microbiome interactions?
- Does *Mtb* infection of APC contribute to a direct or indirect inhibition of ADCC and CTL killing of HIV-1-infected cells?
- Does HIV-1 infection increase *Mtb* dissemination and extrapulmonary latent *Mtb* reservoirs?
- Which cell types are responsible for trafficking and establishing HIV-1 and *Mtb* in tissue reservoirs?
- What is the proportional contribution of macrophage and stem cell reservoir viruses to HIV-1 viral sequences identified in plasma and proximal and distal LN during ART suppression?
- Do individuals on ART who are *Mtb*-infected experience more viral blips once virally suppressed?
- Do individuals who are *Mtb*-infected experience a faster viral rebound following a period of ART withdrawal?

**Executive summary**

**Evolution of the world’s deadliest syndemic infection**

- There was a significantly high incidence of tuberculosis (TB) in sub-Saharan Africa before the HIV-1 epidemic caused up to a tenfold increase in TB incidence. Africa now accounts for 71% of all HIV-TB cases and 84% of coinfection deaths.
- While there has been focused effort on controlling HIV-related TB, there has been less consideration regarding the impact of high community prevalence of latent *Mycobacterium tuberculosis (Mtb)* infection on the HIV-1 epidemic in Africa.

**Acquisition of infection**

- *Mtb* infection creates an expanded cellular niche susceptible to HIV-1 infection, by increasing the density of viral entry coreceptors, CCR5 and CXCR4, on the surface of immune cells.
- Cytokines and chemokines in the proinflammatory microenvironment of *Mtb* infection upregulate HIV-1 replication in bystander cells by activating human transcription factors, which bind to the viral long-terminal repeat promoter.
- Mathematical modeling predicts that coinfected patients have a viral load set point 27% greater than HIV-only infected patients.

**HIV-1 immune evasion & viral spread**

- *Mtb* can exacerbate cell-to-cell HIV-1 transmission by increasing viral transfer between cells, via tunneling nanotubes between macrophages.
- How *Mtb* infection may impair antibody-dependent cellular cytotoxicity and cytolytic T lymphocytes (CTLs)-mediated HIV-1 killing during latent *Mtb* infection is incompletely elucidated.

**Viral reservoirs & latency**

- During early antiretroviral therapy reconstitution, the strongest correlate of restored *Mtb* immunity is an expanded CD27^+^CD4^+^ central memory response, the predominant T-cell reservoir for HIV-1.
- CD16^+^ monocytes that are found in greater frequency in *Mtb* infection, are susceptible to HIV-1 infection and show increased transmigration during HIV-1 infection, increasing the potential intracellular trafficking of HIV-1 to peripheral *Mtb*-infected sites.

**Defining coinfection dynamics in animal models**

- Nonhuman primate (NHP) and bone marrow, liver and thymus (BLT)-humanized mice models allow researchers to build a more detailed understanding of disease progression dynamics, which clinical studies cannot answer.
- Conflicting results of the impact of *Mtb* on simian immunodeficiency virus (SIV)/HIV-1 replication exist between different animal models, which may relate to the diversity and interaction between NHP and pathogens used.
- Tissue level analyses in NHP coinfection provide some evidence that inflammation during TB progression increases SIV replication and integration at the site of disease, with less correlation to total pathogen abundance.
- BLT mice provide evidence that HIV-1 can preferentially replicate within *Mtb* infected lung tissue and there exists increased recruitment of T-cells, which have a higher susceptibility to HIV-1 infection either before or after reaching the *Mtb*-infected lung.

**Implications of latent *Mtb* treatment on HIV-1 prevention & cure strategies**

- ‘Shock and Kill’ is aimed at activating all quiescent HIV-1-infected cells, in order to leverage CTL and antibody-dependent cellular cytotoxicity-mediated killing. The impact of *Mtb* infection on inhibiting CTL targeting of HIV-1 infection is a critical question requiring more research.
- ‘Lock and Block’ aims to prevent HIV-1 from ever being activated. The potential impact of *Mtb* infection activating viral reservoirs during this approach is more evident and should be taken into consideration in future study designs.
